# An Integration of Genome-Wide Association Study and Gene Co-expression Network Analysis Identifies Candidate Genes of Stem Lodging-Related Traits in *Brassica napus*

**DOI:** 10.3389/fpls.2018.00796

**Published:** 2018-06-12

**Authors:** Hongge Li, Xi Cheng, Liping Zhang, Jihong Hu, Fugui Zhang, Biyun Chen, Kun Xu, Guizhen Gao, Hao Li, Lixia Li, Qian Huang, Zaiyun Li, Guixin Yan, Xiaoming Wu

**Affiliations:** ^1^Key Laboratory of Biology and Genetic Improvement of Oil Crops, Oil Crops Research Institute of the Chinese Academy of Agricultural Sciences, Ministry of Agriculture, Wuhan, China; ^2^National Key Laboratory of Crop Genetic Improvement, National Center of Crop Molecular Breeding, National Center of Oil Crop Improvement, College of Plant Science and Technology, Huazhong Agricultural University, Wuhan, China

**Keywords:** *Brassica napus* L., stem lodging-related traits, genome-wide association study, gene co-expression network, candidate genes

## Abstract

Lodging is a persistent problem which severely reduce yield and impair seed quality in rapeseed (*Brassica napus* L.). Enhancing stem strength (SS) has proven to be an effective approach to decrease lodging risk. In the present study, four interrelated stem lodging-related traits, including stem breaking resistance (SBR), stem diameter (SD), SS, and lodging coefficient (LC), were investigated among 472 rapeseed accessions. A genome-wide association study (GWAS) using *Brassica* 60K SNP array for stem lodging-related traits identified 67 significantly associated quantitative trait loci (QTLs) and 71 candidate genes. In parallel, a gene co-expression network based on transcriptome sequencing was constructed. The module associated with cellulose biosynthesis was highlighted. By integrating GWAS and gene co-expression network analysis, some promising candidate genes, such as *ESKIMO1* (*ESK1*, *BnaC08g26920D*), *CELLULOSE SYNTHASE 6* (*CESA6*, *BnaA09g06990D*), and *FRAGILE FIBER 8* (*FRA8*, *BnaC04g39510D*), were prioritized for further research. These findings revealed the genetic basis underlying stem lodging and provided worthwhile QTLs and genes information for genetic improvement of stem lodging resistance in *B. napus*.

## Introduction

Lodging can be defined as the permanent displacement of shoots from their vertical standpoint (Berry, 2013). Lodging is of two types: stem lodging and root lodging, resulting from buckling of the lower stems and failure of the anchorage system, respectively (Berry, 2013). High-yield cultivars accompanying heavy canopy are more prone to stem lodging (Kuai et al., 2016; Kendall et al., 2017). For rapeseed (*Brassica napus* L.), lodging is a persistent problem, leading to 20–46% yield loss and almost 4% oil content reduction during flowering (Kendall et al., 2017) and also impeding crops harvesting (Berry, 2013; Hua et al., 2014). One of the prerequisites for high-yield breeding is to develop stem lodging resistance. Stem lodging risk can be decreased not only by reducing plant height (PH) but also by increasing the physical strength of stem (Shah et al., 2017). However, dwarf/semi-dwarf plant may have limited canopy photosynthesis and biomass production, thereby limiting yield (Stewart et al., 2003; Islam et al., 2007). Thus, the major focus for improving lodging resistance is to enhance stem strength (SS).

In the last decade, enormous efforts were spent on mapping causal loci for lodging-related traits using linkage-based quantitative trait locus (QTL) mapping and linkage disequilibrium (LD)-based genome-wide association study (GWAS). For example, a number of QTLs for lodging-related traits, such as SS and stem diameter (SD), have been identified using linkage mapping in rice (Kashiwagi and Ishimaru, 2004; Dixit et al., 2015), soybean (Chen et al., 2011), and maize (Peiffer et al., 2013). In rapeseed, QTLs for resistant pressure per plant (RPPP), a trait mainly determined by root morphology and stem bending strength (Kashiwagi and Ishimaru, 2004), have been detected using linkage mapping with low-density markers of simple sequence repeat (SSR) and sequence-related amplified polymorphism (SRAP) (Peng, 2012; Li Y. et al., 2014). However, these results are hard to apply in candidate gene characterization because marker information is lacking and QTL regions are still large. GWAS has emerged as an alternative powerful approach for dissecting QTLs that are significantly associated with lodging-related traits in corps, such as in soybean (Wen et al., 2015), wheat (Cericola et al., 2017), and oat (Tumino et al., 2016). In *B. napus*, Wei L. et al. (2017) have identified novel candidate genes for lignin biosynthesis, which is correlated with lodging resistance, by combining GWAS and transcriptome sequencing. However, the causal loci of other characteristics underlying stem lodging, such as xylan and cellulose, have not been well elucidated.

In this study, we investigated four stem lodging-related traits, including stem breaking resistance (SBR), SD, SS, and lodging coefficient (LC) among 472 rapeseed accessions. GWAS of these traits identified 67 significantly associated QTLs and 71 candidate genes. In parallel, a weighted gene co-expression network analysis (WGCNA) based on expression profiles was conducted. This analysis highlighted a module associated with cellulose biosynthesis. By integrating GWAS and WGCNA, we were able to identify novel candidate genes underlying stem lodging resistance in *B. napus*.

## Materials and Methods

### Plant Material and Phenotyping

A population of 472 *B. napus* core germplasm collected worldwide was used for phenotyping in the present study (**Supplementary Table S1**; Li F. et al., 2014). Field experiments were carried out in five environments across three locations and two growing seasons. During the 2014–2015 growing season, the association population was grown at Wuhan (113.68°E, 30.58°N) and Yangluo (114.50°E, 30.38°N), which both in Hubei province of China and were termed as E1 and E2, respectively. During the 2015–2016 cropping season, the experiments were conducted at Wuhan, Yangluo, and Changsha (113.00°E, 28.22°N, in Hunan province of China), which were referred to as E3, E4, and E5, respectively. In each environment, a randomized complete block design with three replicates was implemented. Field cultivation and management were implemented according to previously described method (Chen et al., 2014) and were kept uniform in these five growth environments.

At mid-May, five randomly selected plants from the center of each plot were used to investigate the stem lodging-related traits. SBR of 15 cm length of segment from lower stem was measured using the YYD-1 SS tester (TOP Instrument Co., Zhejiang, China). The maximum strength needed to break the middle of lower stem segment was recorded. SD was represented by measuring the diameter of measurement point of SBR. Then, SS was defined as the SBR on unit area and calculated as follows: SS = SBR/[π(SD/2)^2^]. PH and seed yield (SY) per plant were determined as previously described (Chen et al., 2014). Finally, LC was calculated according to the following formula: LC = PH × SY/SS. The average phenotypic value of five plants in a plot represents the phenotypic data of a line in this plot.

### Statistical Analysis

An lme4 package in R was used to estimate best linear unbiased predictions (BLUPs) across multi-environment on a per line basis for all the lodging-related traits (Merk et al., 2012). The BLUP value and phenotypic data of each accession under single environment were used as the final trait values for GWAS. The broad-sense heritability was estimated according to the following equation: $H^{2}={\text{δ}}_{\text{g}}^{2}/({\text{δ}}_{\text{g}}^{2}+{\text{δ}}_{\text{ge}}^{2}/n\;+\;{\text{δ}}_{\text{e}}^{2}/nr)$, where ${\text{δ}}_{\text{g}}^{2}$ represents the genetic variance, ${\text{δ}}_{\text{ge}}^{2}$ represents the interaction variance between genotypes and environments, ${\text{δ}}_{\text{e}}^{2}$ represents the error variance, *n* represents the number of years/locations, and *r* represents the number of replicates within each environment, respectively. The analysis of variance (ANOVA) of four traits were conducted using “aov” function of R. Correlation matrix was calculated and visualized using corrplot package in R (Wei and Simko, 2016).

### Genotyping With an SNP Array

Single-nucleotide polymorphism (SNP) genotyping had been implemented in previous reports (Li F. et al., 2014; Li et al., 2016). Briefly, genomic DNA of 472 rapeseed accessions was extracted from the young leaf tissue and was then hybridized to the *Brassica* 60K Illumina^®^ Infinium SNP array, which contains 52,157 SNP markers, according to manufacturer’s protocol. The SNPs with minor allele frequency (MAF) < 5% or call frequency <80% were filtered.

In order to mapping SNPs to the physical position of genome, a local BLAST program (Altschul et al., 1990) was performed. The sequences of retained SNPs were queried against the *B*. *napus* genome sequences (Chalhoub et al., 2014). The top and unique blast-hits were selected for the further analysis.

### Population Structure Analysis

We pruned 19,945 SNPs for strong LD (*r*^2^ > 0.8) using PLINK tool (Purcell et al., 2007). These remaining 7458 SNPs were subjected to infer population structure and neighbor-joining (NJ) tree. Population structure was assessed using STRUCTURE software 2.3.4 (Pritchard et al., 2000). The *K*-value (the inferred number of subpopulations) was set from 1 to 10 with five replications under the *admixture* model in the SRUCTURE. The 10,000 iterations were adopted for the burn-in period and the Markov Chain Monte Carlo (MCMC) replications after burn-in. The optimal *K*-value was determined by the *ad hoc* statistic Δ*K*, based on the rate of change in the log probability of data between successive *K*-values (Evanno et al., 2005), using STRUCTURE HARVESTER website^1^ (Earl and Vonholdt, 2012). The STRUCTURE bar plot representing a given *K*-value was obtained with *distruct* (Rosenberg, 2004). In addition, the CLUMPP program was used to align the different replicate runs and integrate a Q-matrix (Jakobsson and Rosenberg, 2007).

An un-rooted NJ phylogenetic tree was constructed using Nei’s genetic distances among 472 lines (Nei, 1972) by using PowerMarker version 3.25 (Liu and Muse, 2005). And the tree was visualized using MEGA7 (Kumar et al., 2016). Principle component analysis (PCA) was conducted with all the SNPs using GCTA tool (Yang et al., 2011) and a P-matrix including the top five principal components was constructed. TASSEL version 5.0 (Bradbury et al., 2007) was used to estimate the relative kinship matrix (K-matrix), which comparing all pairs of the 472 accessions, and the pair-wise LD among all the SNPs, as the squared allele frequency correlations (*r*^2^).

### GWAS and Candidate Gene Selection

Genome-wide association study was implemented using TASSEL 5.0 software (Bradbury et al., 2007) with the Q-model, one of general linear models (GLMs) adjusted using the Q-matrix, and the Q + K model, one of mixed linear models (MLMs) correcting for both Q-matrix and K-matrix. For the GLM, the following equation was used: *y* = *X*β + *e*; and for the MLM, the equation was as follows: *y* = *X*β + *Zu* + *e*, in both equations: *y* is the phenotype, *X* is the genotype, β is a vector containing the fixed effects, *Z* is the relative kinship matrix, *u* is a vector of random additive genetic effects, and *e* is the unobserved vector of the random residual. The significant threshold of SNPs–traits association was set to -log_10_ (*p*) = 4.0. Manhattan plots and quantile–quantile (*Q*–*Q*) plots were produced using the qqman package in R software (Turner, unpublished). The “stepAIC” function from the MASS package in R was employed to estimate the total phenotypic variation explained by the significant SNPs in the best fitting multiple regression model.

The QTL interval where the potential candidate gene situated was determined based on methods of the previous report (Wang et al., 2016). In short, two or more neighboring SNPs within a 1.5-Mb region were defined as one QTL. For the remaining SNP that farther than 1.5 Mb away from another significant SNP, the LD block where the single SNP located, in which flanking markers were in strong LD (*r*^2^ > 0.4), was also regarded as one QTL. An LD block was generated using the Haploview v4.2 (Barrett et al., 2005) and was displayed by LDheatmap package (Shin et al., 2006). A functional annotation was implemented to predict the function of candidate genes using a BlastP program against to *Arabidopsis thaliana* TAIR10 protein database.

### Transcriptome Sequencing Study

To dissect the expression pattern of stem lodging-related genes during flowering and silique developing, the stems from two extremely high-SBR lines (3260 and 5721) and two extremely low-SBR lines (3399 and 3453) were harvested and pooled as four independent samples that were designated as FH (High-SBR during Flowering), FL (Low-SBR during Flowering), SH (High-SBR during Silique developing), and SL (Low-SBR during Silique developing), respectively. Total RNA was extracted using SV Total RNA Isolation System (Promega Biotech Co., Ltd., Beijing, China). After a test of total RNA quality, the 12 cDNA libraries of four samples (three biological replicates per sample) were constructed. Subsequently, these libraries were sequenced using an Illumina HiSeq X Ten sequencer (Illumina Inc., San Diego, CA, United States), and millions of 150 bp paired-end reads were generated.

The clean reads were obtained by trimming the raw data. The clean data were deposited in the Short Read Archive (SRA) database of NCBI under the accession number SRP142441. These clean reads were aligned to the *B*. *napus* reference genome (Chalhoub et al., 2014) using HISAT v2.0.4 (Kim et al., 2015). To quantify the gene expression level, HTSeq v0.6.1 was used to count the FPKM values (the number of fragments per kilobase of transcript sequence per millions mapped reads, Anders et al., 2015). The criteria|log_2_ (High-SBR/Low-SBR)| > 1 and *P* < 0.05 was applied here to identify differentially expressed genes (DEGs) between two genotypes.

### Construction of Gene Co-expression Network

Weighted correlation network analysis was conducted using WGCNA version 1.61 package in R software (Langfelder and Horvath, 2008). A total of 8850 genes with FPKM value > 3 and coefficient of variation of FPKM higher than 0.3 across all samples were included in the WGCNA workflow. In WGCNA, the scale-free topology criterion was used to determine the soft-threshold, which is defined as the similarity relationships between gene-pairs and obtained by computing the unsigned Pearson’s correlation matrix (Zhang and Horvath, 2005). Subsequently, network was constructed using a step-by-step method by turning adjacency matrix into topological overlap matrix (TOM) and calling the hierarchical clustering function. Module identification was implemented after merging of modules whose expression profiles are very similar with a merge CutHeight of 0.25. The eigengene values of all detected 25 modules were calculated to assess module-sample relationships. To explore the biological meaning of each module, gene ontology (GO) enrichment analysis was performed using the OmicShare tools, a free online platform for data analysis^2^. The interaction network of hub-genes in module was visualized using Cytoscape 3.5.1 (Shannon et al., 2003).

### Validation of DEGs by qRT-PCR

The expression level of DEGs was validated by qRT-PCR as described previously (Yan et al., 2016). In brief, RNA samples prepared for transcriptome sequencing were used for cDNA synthesis; expression of the DEGs in different rapeseed samples was evaluated using LightCycler 480 SYBR Green I Master mix and a LightCycler 480II real-time PCR system (Roche, Switzerland). *Bna.ACTIN7* and *Bna.UBC21* were included as housekeeping genes and relative expression levels of target genes were normalized to geometric mean of two housekeeping genes using the 2^-ΔΔC_T_^ method. Gene-specific primers were listed in **Supplementary Table S8**. Three independent biological replicates, each with three technical replicates, were run for target and housekeeping genes.

## Results

### Phenotypic Variation and Correlation Analysis

Four stem lodging-related traits across 472 accessions in five environments (LC in four environments) were evaluated and extensive phenotype variations were found (**Table 1** and **Supplementary Figure S1**). The SBR was varied from 6.45 to 153.56 N with an average of 51.94 N, the SD was varied from 4.68 to 17.11 mm with an average of 10.77 mm, the SS was varied from 0.20 to 1.11 N/mm^2^ with an average of 0.54 N/mm^2^, and the LC was varied from 407.66 to 8461.42 with an average of 2569.71, respectively (**Table 1**). An ANOVA of these traits revealed significant differences among genotypes, environments, and an interaction between genotype and environment (*P* < 0.001, **Supplementary Table S2**). The broad-sense heritability (*H*^2^) of SBR, SD, SS, and LC in the rapeseed population was 67.46, 61.30, 66.54, and 51.85%, respectively (**Supplementary Table S2**), suggesting that environmental factors had limited influence on these traits.

**Table 1:** Phenotypic variation for stem lodging-related traits among 472 accessions.

Traits	Environments	Min	Max	Mean	SD (%)^a^	CV^b^
**SBR**	E1	24.22	153.56	68.10	21.56	0.32
	E2	16.60	138.13	56.11	16.24	0.29
	E3	22.31	151.54	63.26	21.82	0.34
	E4	13.60	150.78	48.89	16.32	0.33
	E5	6.45	66.56	23.28	8.27	0.36
**SD**	E1	8.17	15.45	11.63	1.24	0.11
	E2	6.25	14.78	11.75	1.20	0.10
	E3	7.78	17.11	11.82	1.51	0.13
	E4	6.98	15.24	11.25	1.30	0.12
	E5	4.68	10.97	7.43	1.06	0.14
**SS**	E1	0.37	1.11	0.62	0.11	0.18
	E2	0.29	0.96	0.50	0.09	0.19
	E3	0.20	1.02	0.56	0.12	0.21
	E4	0.21	0.93	0.48	0.10	0.21
	E5	0.26	0.90	0.52	0.10	0.19
**LC**	E1	∖				
	E2	1073.37	8275.52	3546.34	1018.02	0.29
	E3	433.72	8461.42	2396.65	1067.23	0.45
	E4	728.79	7926.33	3052.70	1130.86	0.37
	E5	407.66	3567.37	1283.13	436.52	0.34

*SBR, stem breaking resistance (N); SD, stem diameter (mm); SS, stem strength (N/mm^2^); LC, lodging coefficient; E1, Wuhan in 2015; E2, Yangluo in 2015; E3, Wuhan in 2016; E4, Yangluo in 2016; and E5, Changsha in 2016. ^a^Standard deviation. ^b^Coefficient of variation.*

**FIGURE 1:**
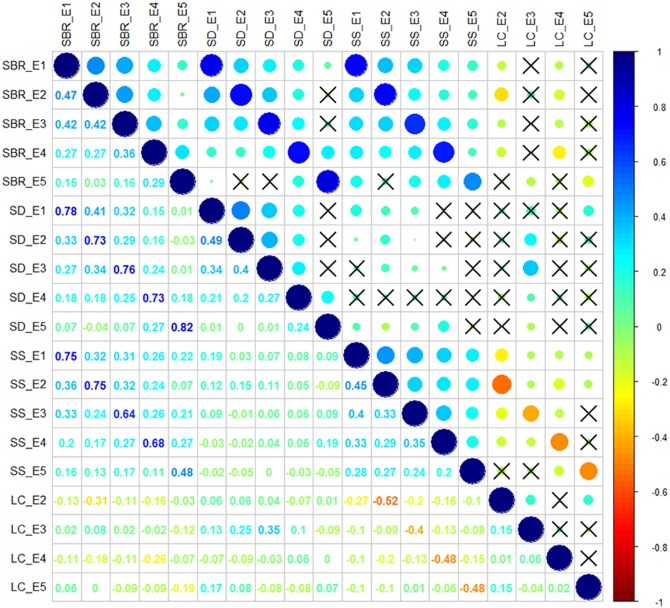
Correlation analysis among stem lodging-related traits. SBR, stem breaking resistance (N); SD, stem diameter (mm); SS, stem strength (N/mm^2^); LC, lodging coefficient; E1, Wuhan in 2015; E2, Yangluo in 2015; E3, Wuhan in 2016; E4, Yangluo in 2016; and E5, Changsha in 2016. The magnitude of correlation is indicated by different colors of number at the left diagonal and circles at the right diagonal. The circles marked by cross indicate no significantly correction was observed between traits (*P* > 0.05).

Correlation analysis of the traits showed that SBR was positively correlated with SD (*r* = 0.73–0.82, *P* < 0.05) and SS (*r* = 0.48–0.75, *P* < 0.05) across each environment. While the negative correlations were observed between SS and LC (*r* = -0.52 to -0.40, *P* < 0.05) across each environment (**Figure 1**). The interrelationship between traits illustrated that it is essential to take the multiple interrelated traits into account to comprehensively assess stem lodging resistance performance.

### Population Structure and Relative Kinship Analysis

A final set of 19,945 high-quality SNPs distributed on the whole genome was subjected to PCA, and relative kinship analysis. After pruning the 19,945 SNPs for strong LD (*r*^2^ > 0.8), 7458 SNPs were applied to infer population structure and NJ tree.

**FIGURE 2:**
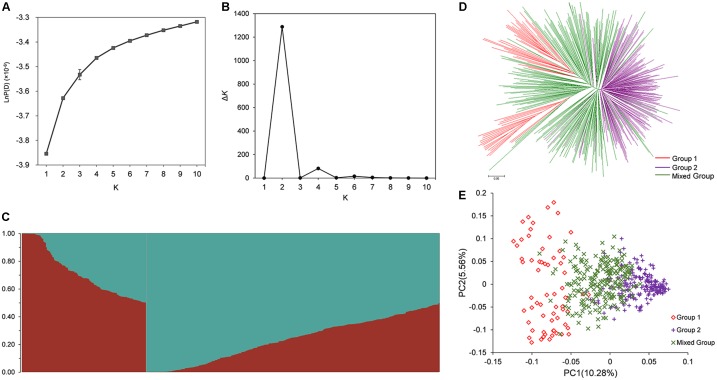
Population structure analysis of association population in rapeseed. **(A)** The variation trend of LnP(*K*) with change of *K*-value from 1 to 10. **(B)** Delta *K* based on the rate of change of LnP(*K*) between successive *K*. **(C)** Population structure defined by STRUCTURE when *K* = 2. **(D)** NJ tree analysis based on 472 rapeseed accessions. **(E)** Principal component analysis based on 472 rapeseed accessions. 472 lines were clustered into Group 1, Group 2, and Mixed Group based on the Δ*K-*value and the probability of membership.

The population structure across 472 rapeseed accessions was investigated using STRUCTURE software. The LnP (*K*) values gradually increased with *K*-value from 1 to 10 (**Figure 2A**); however, the summit of Δ*K* value was achieved at *K* = 2 (**Figure 2B**). These data in **Figure 2B** implied that 472 lines could be clustered into two groups (**Figure 2C**). By applying a membership threshold of 0.70, the first group of 64 and the second group of 199 accessions were assigned as Group 1 and Group 2, respectively. The remaining 209 accessions were assigned into a Mixed Group. The assignment of groups for all the accessions was presented in **Supplementary Table S1**. To dissect the genetic structure of the association population, molecular phylogenetic analysis based on the Nei’s genetic distances of 7458 SNPs was conducted. The NJ tree showed that the accessions displayed three divergent groups that mostly consistent with the groups assigned by STRUCTURE (**Figure 2D**). However, the Group 1 was divided into two sub-clusters. Further analysis for the two sub-clusters in Group 1 showed most of the lines in one of the sub-clusters were winter rapeseed (36 out of 37 lines), while approximately 75% (20 out of 27 lines) of the other sub-cluster were spring rapeseed. In Group 2, most of lines were from Asia, and 129 lines from China were semi-winter type. In the Mixed Group, winter rapeseed (83 lines) were the largest part, followed by semi-winter rapeseed (72) and spring rapeseed (53) (**Supplementary Table S1**). A similar pattern of grouping was also revealed through PCA (**Figure 2E**). The first two principal components explained 10.28 and 5.56% of the genetic variance, respectively (**Figure 2E**).

In addition, the relative kinship analysis indicated that average kinship coefficient between any two accessions was 0.097. A total of 52.5% of pairwise kinship coefficients were equal to 0, and 63.5% of kinship coefficients varied from 0 to 0.05 (**Supplementary Figure S2**).

### Genome-Wide Association Study

**FIGURE 3:**
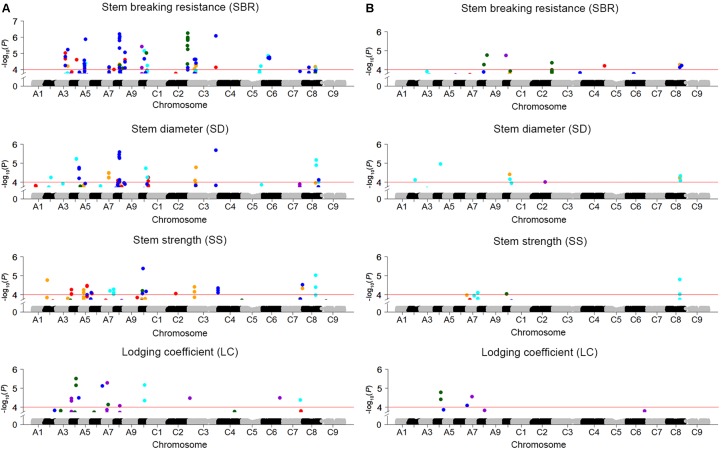
Manhattan plots for GWAS results of stem lodging-related traits in five environments and BLUP. **(A)** SBR, SD, SS, and LC in GLM. **(B)** SBR, SD, SS, and LC in MLM. The horizontal red line indicates the significance threshold [–log_10_ (*p*) = 4.0]. The orange, red, cyan, dark violet, dark green, and blue dots above red line represent SNPs significantly associated with traits in E1, E2, E3, E4, E5, and BLUP, respectively.

Genome-wide association study was conducted to explore the association between SNPs and four stem lodging-related traits across 472 rapeseed accessions. A total of 158 significantly associated SNPs have been identified using the BLUP value and individual environment phenotypic value in GLM and MLM, respectively (**Figure 3** and **Supplementary Table S3**). These SNPs were located on all the chromosomes except A1 and C9. Moreover, 34 SNPs were detected repeatedly across multiple environments (**Supplementary Table S3**).

For SBR, altogether 78 significantly associated SNPs, which collectively explained 38.1% of the total phenotypic variation and corresponded to 26 QTLs, were identified in GLM and MLM (**Supplementary Tables S3**, **S4**). Half of loci were verified in at least two environments, and three loci (*SBR-A08c*, *SBR-A10a*, and *SBR-C08b*) were discovered repeatedly both in GLM and MLM. In addition, the locus *SBR-A08a* in A08 was stably expressed across five environments (**Supplementary Table S4**). For SD and SS, 31 and 35 significantly associated SNPs, which contributed to 23.7 and 23.3% of the cumulative phenotypic variation and were assigned to 16 and 15 QTLs, were detected in GLM and MLM, respectively (**Supplementary Tables S3**, **S4**). For LC, a total of 14 significantly associated SNPs corresponding to 10 loci, which explained 4.12–6.40% of the phenotypic variation, were detected (**Supplementary Tables S3**, **S4**).

**FIGURE 4:**
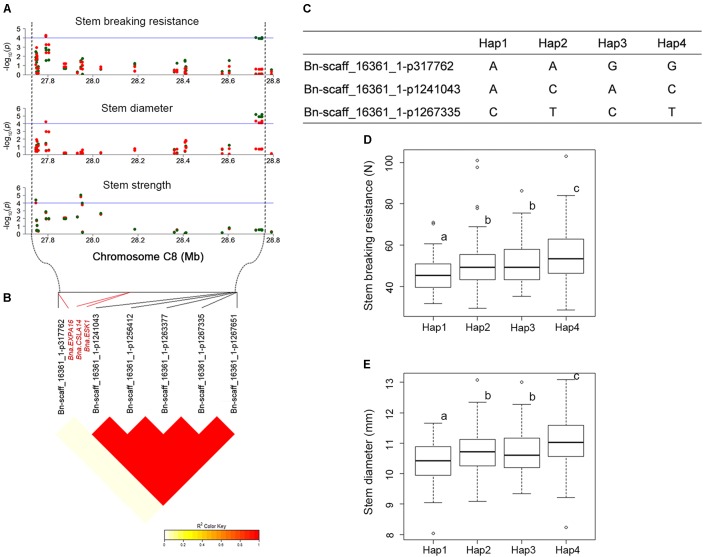
LD and haplotype (Hap) effect of significant SNPs on overlapped loci on C08. **(A)** Manhattan plots for QTLs (*SBR-C08b*, *SD-C08a*, and *SS-C08b*) on C08 chromosomal region for SBR, SD, and SS, respectively. The horizontal blue line indicates the significance threshold [–log_10_ (*p*) = 4.0]. The green and red dots represent SNPs detected in GLM and MLM, respectively. **(B)** LD plot of significant SNPs. **(C)** Haps analysis based on three overlapped significant SNPs between SBR and SD. **(D)** Hap effect for SBR. **(E)** Hap effect for SD. Statistical significance was determined with ANOVA, different letters (a, b, and c) represent significant difference at 5% level.

QTL that control more than one trait are of special interest to us. For instance, *SBR-C08b*, a locus for SBR, which across from 27,791,286 to 28,749,469 on C08, was consistently detected on the corresponding vicinity region for the traits of SD (*SD-C08a*, spanning from 27,791,286 to 28,749,785) and SS (*SS-C08b*, spanning from 27,604,355 to 27,952,594). What’s more, this locus has been identified by using both GLM and MLMs for these three traits (**Figures 3**, **4A** and **Supplementary Table S4**). Thus, we analyzed the LD and haplotype (Hap) effect of significant SNPs on this QTL on C08. A high LD between the associated SNPs was observed (**Figure 4B**). We detected four major Haps based on the three shared significant SNPs that detected in both SBR and SD among the 472 rapeseed accessions (**Figure 4C**). The average SBR value of Hap4 was remarkably higher than those in the other three Haps (**Figure 4D**, *P* < 0.05). The comparative analysis of SD between Haps exhibited a similar difference pattern (**Figure 4E**, *P* < 0.05).

### Transcriptome Sequencing Analysis

For these four samples (FH, FL, SH, and SL), a total of 146.35, 156.91, 158.59, and 143.76 million raw reads were generated (**Supplementary Table S5**). After removing low quality reads and adaptor sequences, clean reads with an average of 50.28 million reads per sample were obtained. Moreover, approximately 80% of clean reads were uniquely mapped to *B. napus* reference genome (**Supplementary Table S5**). Of the DEGs identified between high-SBR and low-SBR, 1679 DEGs were up-regulated and 2,586 DEGs were down-regulated during flowering, while 706 DEGs were up-regulated and 414 DEGs were down-regulated during silique developing (**Supplementary Figure S3**). Moreover, 361 common up-regulated DEGs and 253 common down-regulated were identified between two development stages (**Supplementary Figure S3**).

### Gene Co-expression Network Construction and Analysis

**FIGURE 5:**
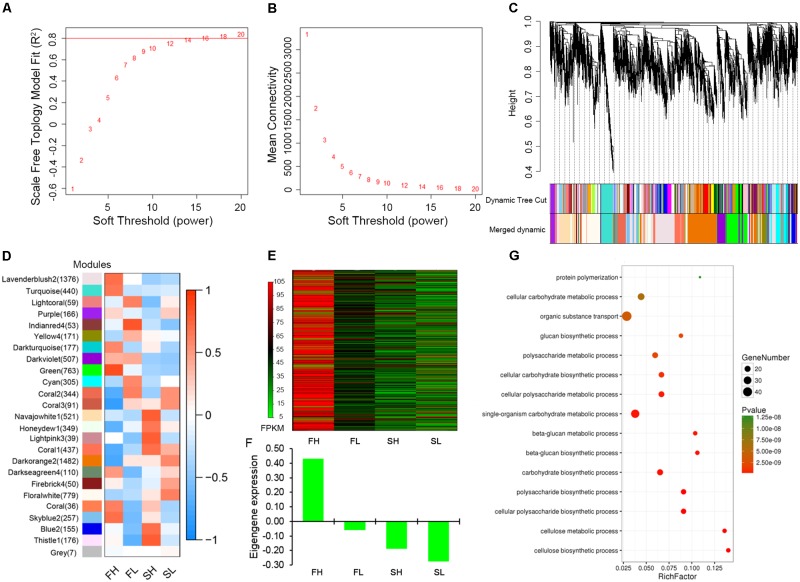
Gene co-expression network construction and “green” module analysis. **(A)** Estimated scale-free topology fit value of power (β) from 1 to 20. **(B)** Mean connectively between successive powers. **(C)** Cluster dendrogram showing co-expression modules. Each leaf in the tree is one gene and modules corresponding to branches are labeled by different colors. **(D)** Module–sample association. Each row corresponds to a module and each column corresponds to a sample. The number in brackets indicates the number of genes in each module. **(E)** Heat map illustrating the FPKM values of 763 genes in “green” module. **(F)** The eigengene expression analysis for “green” module. **(G)** GO enrichment analysis in biological processes for “green” module. FH, High-SBR during Flowering; FL, Low-SBR during Flowering; SH, High-SBR during Silique developing; SL, Low-SBR during Silique developing.

Weighted gene co-expression network analysis was performed to construct the undirected and weighted gene networks and detect the modules related to stem lodging-related traits. When power β = 14, the scale-free topology fit does not improve after increasing the power (**Figure 5A**). Furthermore, at this power, a high mean number of connections was maintained (**Figure 5B**), suggesting that power β = 14 is a proper parameter to construct network. The highly interconnected genes are then clustered into a module. In this study, 8,850 genes were assigned into 24 distinct modules and 7 genes were not assigned into any module, which is denoted as “gray” module (**Figure 5C** and **Supplementary Table S6**).

To represent gene expression profiles of a module, module eigengene (ME), the first principal component of a given module, has been calculated. The correlations between MEs and specific samples were shown in **Figure 5D**. A high positive correlation was found between FH sample and “green” module which includes 763 genes, while the negative correlations were found between the other three samples and “green” module (**Figure 5D**). These data imply that genes enriched in this module might play important roles in lodging resistance regulation in rapeseed and we put our focus on this module for further exploration.

### “Green” Module Was Associated With Cellulose Biosynthesis

By comparing the FPKM values of 763 genes in the “green” module across four samples (FH, FL, SH, and SL) in a heatmap, we revealed that most of the genes enriched in the “green” module were highly expressed in FH sample and weakly expressed or almost not expressed in the other three samples (**Figure 5E**). And the eigengene expression analysis for “green” module among four samples displayed the similar expression trend (**Figure 5F**). To provide biological interpretation of the gene network in “green” module, enrichment analyses of GO terms were conducted. For Biological Processes ontology, the genes in “green” module are mostly enriched for cellulose biosynthetic process (GO:0030244), cellulose metabolic process (GO:0030243), glucan biosynthetic, and metabolic process (GO:0051274, GO:0051273, and GO:0009250) (**Figure 5G**). For Molecular Function ontology, the most prevalent GO terms were cellulose synthase activity (GO:0016759 and GO:0016760) and [uridine diphosphate (UDP)-] glucosyltransferase activity (GO:0035251 and GO:0046527; **Supplementary Figure S4**). Collectively, these genes in the “green” module mainly played roles during flowering and were mainly enriched in function of cellulose biogenesis and metabolic process.

**FIGURE 6:**
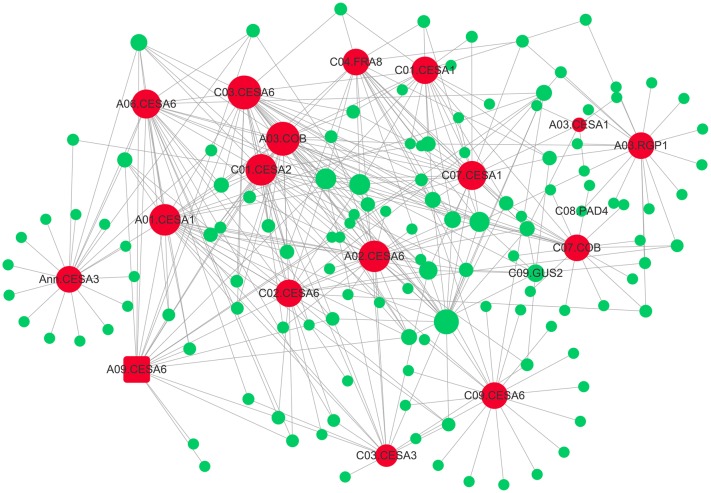
The gene co-expression network of “green” module. The 17 genes involving in cellulose biosynthesis are indicated by larger red circles and square (*A03*.*CESA6*), respectively. The 17 hub-genes’ top20 genes in weight values were colored by green.

Subsequently, the interaction network between cellulose biosynthesis genes and theirs top20 genes in weight values was constructed using Cytoscape (**Figure 6**). The network showed pivotal roles of *FRAGILE FIBER 8* (*FRA8*), two *COBRA* (*COB*), *REVERSIBLY GLYCOSYLATED POLYPEPTIDE 1* (*RGP1*), and 13 primary cell wall *CELLULOSE SYNTHASEs* (*CESA*s), including four *CESA1*, one *CESA2*, two *CESA3*, and six *CESA6* (**Figure 6**). The mutation of primary *CESA*s, *FRA8*, and *RGP1* lead to cellulose defect in the cell wall of *A. thaliana* (Zhong et al., 2005; Rautengarten et al., 2011; Kumar and Turner, 2015).

### Candidate Genes Prediction and Prioritization by Integrating GWAS and Gene Co-expression Network

The potential candidate genes within the interval of QTLs were predicted by blasting against *Arabidopsis* gene database. In total, 71 candidate genes have been identified for stem lodging-related traits in GWAS. Among these genes, 24, 17, 14, and 17 were localized within the search window of 10, 10, 8, and 6 loci for trait SBR, SD, SS, and LC, respectively (**Table 2** and **Supplementary Table S7**).

**Table 2:** A list of priority candidate genes for stem lodging-related traits.

Candidate genes	Gene name	Homolog	Gene function	Trait
*BnaA03g47330D*	*TBL18*	*AT4G25360.2*	Encodes a member of the TBL gene family. Involves in the synthesis and deposition of secondary wall cellulose.	SBR
*BnaA05g15310D*	*4CL1*	*AT1G51680.1*	Encodes an isoform of 4-coumarate:coa ligase, which is involved in the last step of the general phenylpropanoid pathway.	SBR
*BnaA05g16350D*	*CSLD6*	*AT1G32180.1*	Encodes a gene similar to cellulose synthase.	SBR
*BnaA08g11640D*	*CCoAOMT1*	*AT4G34050.1*	Caffeoyl-coa *O*-methyltransferase activity, involves in coumarin biosynthetic process.	SBR
*BnaA09g06870D*	*XND1*	*AT5G64530.1*	Involves in xylem development.	SBR
*BnaA09g06990D*^a^	*CESA6*	*AT5G64740.1*	Encodes a cellulose synthase isomer. Involves in primary cell wall biogenesis.	SBR
*BnaA10g24260D*	*TBR*	*AT5G06700.1*	Encodes a member of the TBL gene family. Involves in the synthesis and deposition of secondary wall cellulose.	SBR
*BnaC04g47040D*	*TBL33*	*AT2G40320.1*	Encodes a member of the TBL gene family. Involves in the synthesis and deposition of secondary wall cellulose.	SBR
*BnaC08g26400D*	*EXPA16*	*AT3G55500.1*	Involves in plant-type cell wall loosening, modification, multidimensional cell growth, and organization.	SBR, SD, SS
*BnaC08g26920D*	*ESK1*	*AT3G55990.1*	Encodes a member of the TBL gene family. Involves in the synthesis and deposition of secondary wall cellulose, xylan biosynthetic process.	SBR, SD
*BnaC08g26930D*	*CSLA14*	*AT3G56000.1*	Encodes a gene similar to cellulose synthase.	SBR, SD
*BnaA08g07050D*	*BRX*	*AT1G31880.1*	Encodes a key regulator of cell proliferation and elongation in the root.	SD
*BnaA05g14370D*^b^	*ARF2*	*AT5G62000.3*	Encodes an auxin response factor.	SS
*BnaC02g23170D*	*FRA2*	*AT1G80350.1*	Involves multidimensional cell growth, plant-type cell wall biogenesis.	SS
*BnaC03g22200D*	*EXPA4*	*AT2G39700.1*	Involves in plant-type cell wall loosening, modification, multidimensional cell growth, and organization.	SS
*BnaA04g03540D*	*EXPA16*	*AT3G55500.1*	Involves in plant-type cell wall loosening, modification, multidimensional cell growth, and organization.	LC
*BnaA07g10240D*	*CEL5*	*AT1G22880.1*	Involves in cell wall organization, cellulose catabolic process	LC
*BnaA07g12000D*	*IRX14L*	*AT5G67230.1*	Encodes a member of the GT43 family glycosyltransferases. Involves in cell wall organization, glucuronoxylan biosynthetic process, plant-type secondary cell wall biogenesis.	LC
*BnaC04g39510D*^c^	*FRA8*	*AT2G28110.1*	A member of glycosyltransferase family 47 that is involved in secondary cell wall biosynthesis.	

*SBR, stem breaking resistance; SD, stem diameter; SS, stem strength; LC, lodging coefficient. ^a^This gene was identified using GWAS and gene co-expression network. ^b^This gene was identified using GWAS, it was also differentially expressed between high-SBR and low-SBR. ^c^This gene was only identified using gene co-expression network.*

We consider candidate genes that have been identified on overlapped QTL as most promising candidates for future studies. At the above-mentioned hot spot on C08 (*SBR-C08b*, *SD-C08a*, and *SS-C08b*), three promising candidate genes, *ESKIMO1* (*ESK1*, *BnaC08g26920D*), *CELLULOSE SYNTHASE LIKE A14* (*CSLA14*, *BnaC08g26930D*), and *EXPANSIN A16* (*EXPA16*, *BnaC08g26400D*), have been identified in both models (**Figure 4B**, **Table 2**, and **Supplementary Table S7**). *BnaC08g26920D*, the ortholog of *ESK1*/*TRICHOME BIREFRINGENCE-LIKE 29* and a member of the *TBL* gene family, was detected at 28.1 Mb on C08 for SBR and SD. *ESK1* plays an essential role in xylan *O*-acetylation during secondary wall biosynthesis in *Arabidopsis* (Yuan et al., 2013). In this overlapped QTL, on 3.7 kb downstream from *BnaC08g26920D* (*ESK1*), the ortholog of *CSLA14*, *BnaC08g26930D*, was a member of cellulose synthase-like gene family and was detected for SBR and SD. In addition, the other genes involved in cellulose biosynthesis have also been identified, such as *BnaA09g06990D* for SBR, the ortholog of *CESA6* (**Table 2**; Fagard et al., 2000). Besides the orthologs of *ESK1* and *CSLA14*, *BnaC08g26400D*, which is ortholog of *EXPA16*, has also been identified in the same QTL for the trait SBR. The *EXPA16* has been reported to be involved in multidimensional cell growth and cell wall loosening (Wieczorek et al., 2006). Interestingly, this gene is also a candidate gene for traits of SD and SS.

More interestingly, among these 71 candidates identified in GWAS, five genes (*BnaA02g12730D*, *BnaA05g14370D*, *BnaA07g11030D*, *BnaC01g01290D*, and *BnaC07g40400D*) were differentially expressed between high-SBR and low-SBR (**Supplementary Table S7**). It is remarkable that, *BnaA05g14370D*, the ortholog of *AUXIN RESPONSE FACTOR 2* (*ARF2*) was continuously down-regulated during flowering and silique developing (**Supplementary Table S7**). It has been reported that *arf2* mutants exhibit thick and long inflorescence, implying a function of SS regulation for *ARF2* (Okushima et al., 2005).

Considering that the genes in “green” module were mainly enriched in function of cellulose biogenesis process and our GWAS candidate list also includes a cellulose biosynthetic gene, thus, a novel candidate gene, *CESA6* (*BnaA09g06990D*), was identified jointing GWAS and gene co-expression network (**Table 2**). *CESA6* encodes a cellulose synthase isomer and its mutant has cellulose defect in the primary cell wall and shows a short-hypocotyl phenotype in *Arabidopsis* (Fagard et al., 2000; Hématy et al., 2007). In addition, although *FRA8* (*BnaC04g39510D*) was only identified in gene co-expression network (**Table 2**), it was also a high-promising candidate gene in virtue of exactly and dramatic mutants phenotype in *Arabidopsis* (Zhong et al., 2005).

### DEGs Validation

The five DEGs (*BnaA02g12730D*, *BnaA05g14370D*, *BnaA07g11030D*, *BnaC01g01290D*, and *BnaC07g40400D*) identified in GWAS were selected to validate the gene expression patterns by qRT-PCR. The expression patterns of all five genes were consistent with those showed in RNA-seq (**Supplementary Figure S5**), proving the reliable of our data.

## Discussion

### Population Structure in Association Mapping Population

The spurious association between traits and markers would emerge when population structure was ignored. Hence, to reduce both Type I and II errors (false positives and false negatives), it is essential to deepen the understanding of population structure in rapeseed. In this study, when *K* = 2, the highest Δ*K* value was observed (**Figure 2B**), meaning that the association population could be classified into two subpopulations. However, this result is not in accordance with previously released report (Li F. et al., 2014). In which, the same population was used for association mapping, however, the elbow of Δ*K* emerged at *K* = 3. This difference might be caused by two reasons: first, the position of SNPs in that paper was obtained by mapping the SNPs of array to “pseudomolecules” representative of the *B*. *napus* genome. Because the *B*. *napus* reference genome (Darmor v4.1) had not been released at that time (Chalhoub et al., 2014), all the SNPs position were hypothetical; second, the SNPs used for population structure analysis in the present study was almost double as much as that study (Li F. et al., 2014). All these might affect the analysis outcomes of population structure which generated by STRUCTURE software.

In our study, 472 rapeseed accessions could be clustered into three groups when the probability of membership threshold was considered (**Figures 2D,E** and **Supplementary Table S1**). However, Group 1 was divided into two sub-clusters, which mainly consisted of winter rapeseed and spring rapeseed, respectively, by the lines belong to Mixed Group (**Figures 2D,E**). The component of Group 2 demonstrated the similar phenomenon, that is, association panels are difficult to completely be separated according to ecotype or geographical origin. Moreover, the lines in Mixed Group often located at an intermediate position between Groups 1 and 2. This phenomenon has been reported in previous studies (Li F. et al., 2014; Xu et al., 2016) and could possibly be interpreted by the introgression induced by interspecific hybridization between diverse germplasms. When winter rapeseed originated from Europe was introduced to other countries, including China, Australia, and Canada, interspecific hybridization between different gene pools was carried out to improve adaptation and quality, accompanying with the generation of semi-winter rapeseed and spring rapeseed (Wei D. et al., 2017).

### An Integration of GWAS and WGCNA Provides a Powerful Approach for Candidate Gene Prediction

Recently, GWAS has been widely applied to identify QTLs and thus shed light on the genetic basis underlying important agronomic traits in *B*. *napus*. However, there are still many obstacles in the fine mapping and target genes cloning due to the large region of the identified QTLs and lack of comprehensive understanding of the genetic mechanisms of complex traits. In the face of these challenges, a very promising strategy by combing both GWAS and RNA-seq has been employed to functionally characterize the association results. By using this approach, the key genes associated with agronomic traits such as fiber developmental traits in upland cotton and resistance to *Sclerotinia* stem rot and lodging in rapeseed have been discovered (Sun et al., 2017; Wei et al., 2016; Wei L. et al., 2017). However, a particular phenotype is a reflection of network effect of interrelated genes (Kobayashi et al., 2016). The RNA-seq approach based on gene expression changes in those studies may not effectively reflect the regulatory network of traits because of the absence of information about interrelationship of genes. To solve this problem, WGCNA serves as a systems biology method to help interpret the correlation patterns between genes, thus facilitating our understanding of gene networks instead of individual genes (Langfelder and Horvath, 2008). In *Arabidopsis*, for instance, the combined use of GWAS and WGCNA has already been utilized to exploit candidate genes that are likely associated with salt tolerance (Kobayashi et al., 2016).

In this study, we are the first to combine both GWAS and WGCNA to excavate candidate genes underlying complex trait in *B. napus*. As a result, the significant candidate genes regulating stem lodging, such as *ESK1*, *CESA6*, and *FRA8*, were identified (**Table 2**).

### Stem Lodging Resistance in Rapeseed

Lodging is causing tremendous yield and quality reduction, accordingly, it is imperative to deepen understanding to lodging resistance in crops. However, lodging is a complex trait which involves traits of SS, SD, canopy structure, and so on, and is affected by genetic, husbandry, and environmental factors (Berry, 2013; Baker et al., 2014).

According to previous studies in *Arabidopsis* and rice, many genes involved in lodging resistance have been identified, such as *CINNAMYL ALCOHOL DEHYDROGENASE*-*C* (*CAD-C*) and *CAD-D*, that act as the primary genes involved in lignin biosynthesis in *Arabidopsis* (Sibout et al., 2005); *CESA4* and *CESA9*, that are involved in cellulose biosynthesis in rice (Zhang et al., 2009; Li et al., 2017); *REDUCED WALL ACETYLATION 1*/*2*/*3*/*4* (*RWA1*/*2*/*3*/*4*), that are required for the acetylation of xylan in *Arabidopsis* (Lee et al., 2011); *IRREGULAR XYLEM 9* (*IRX9*), *IRX9L*, and *IRX14*, that are from glycosyltransferase family and play roles in xylan biosynthesis in rice (Chiniquy et al., 2013). However, there is still limited study in lodging resistance in *B. napus*. To date, little of genes regulating stem lodging have been identified. Wei L. et al. (2017) identified four candidate genes regulating lignin. However, the genes controlling other chemical composition of cell wall, such as xylan and cellulose, have not been included in that paper.

The stem physical strength is mainly maintained by cell wall development. Plant cell wall can be roughly characterized into two types: the primary cell wall, which is composed of cellulose, hemicellulose, and pectin, and the secondary cell wall, which is composed of cellulose, hemicellulose, and lignin (Li S. et al., 2014). These polymers of cell wall provide a framework that gives further strength to cells that have to sustain enhanced mechanical stress (Bischoff et al., 2010).

In our study, we identified candidate genes regulating xylan and cellulose. Xylan is one component of hemicelluloses, whose acetylation could provide plants mechanical strength (Urbanowicz et al., 2014). *ESK1*/*TBL29* (*BnaC08g26920D*) coding xylan *O*-acetyltransferases have been identified for SBR and SD in our GWAS result (**Table 2** and **Supplementary Table S7**). *ESK1*/*TBL29* has been reported to play an essential role in xylan *O*-acetylation during secondary wall biosynthesis. The *esk1* mutation exhibited collapsed xylem as well as reduced secondary wall thickening and attenuated stem mechanical strength (Lefebvre et al., 2011; Yuan et al., 2013, 2016).

Another candidate gene, *FRA8/IRREGULAR XYLEM 7*(*IRX7*) gene, has also been reported to be involved in xylan biosynthesis (Zhong et al., 2005). The *Arabidopsis FRA8* mutation caused a dramatic reduction in xylan content and fiber wall thickness as well as a decrease in cellulose content and SS (Zhong et al., 2005). Notably, it is known that variation in stem tensile strength is associated with variation in fiber dimensions, such as cell wall thickness and fiber length. Recently, a strong candidate gene *FRA8* for fiber length was identified using association mapping in *Populus* (Porth et al., 2013). In the present study, *FRA8* (*BnaC04g39510D*) was identified as the hub-gene in “green” module, which was associated with cellulose biosynthesis, using WGCNA (**Table 2** and **Figure 6**). In addition, although *FRA8* (*BnaC04g39510D*) was not mined as a candidate gene using association mapping here, its duplicated gene, *FRA8* (*BnaA04g16260D*) was identified as highly promising candidate gene for trait of SBR in our another association mapping based on re-sequencing (data not shown), which implied that *FRA8* possibly plays a key role in stem lodging regulation in rapeseed.

In addition, we also found a promising candidate gene involved in cellulose biosynthesis, which is vital for cell wall development. By association mapping, we have identified the candidate gene *CESA6* (*BnaA09g06990D*) for SBR (**Table 2** and **Supplementary Table S7**). *CESA6* is 431.8 Kb away from significant SNP Bn-A09-p3051349, which was also detected for SBR in previously report (Wei L. et al., 2017) and was the only one overlapped SNP between that report and our study. Moreover, *CESA6* (*BnaA09g06990D*) was also identified in “green” module using WGCNA. In higher plants, cellulose, which consists of β-1,4-linked glucan chains, is synthesized by CESA complexes (CSC) using UDP-glucose as substrates (Somerville, 2006). *CESA6* is a member of CSC that synthesize primary cell wall cellulose. The strong cellulose deficiency and short hypocotyl phenotypes were observed in mutants of *CESA6* (Fagard et al., 2000; Hématy et al., 2007). Furthermore, for *CESA6*, besides a role in primary wall development, it may also continue to transiently express during secondary cell wall development in aspen trees (Samuga and Joshi, 2004).

Although a better understanding for stem lodging was acquired with the identification of potential associated loci and promising candidate genes of stem lodging-related traits, the genetic regulation mechanism, especially gene regulation network, underlying stem lodging remains elusive. Therefore, more thorough research is still required to elucidate the molecular basis of stem lodging regulation and lay the foundation for genetic improvement of lodging resistance in *B. napus*.

## Author Contributions

HoL, GY, and XW conceived and designed the study. BC, KX, and GG organized the implementation of field trials. LZ, FZ, HaL, LL, and QH performed the phenotyping measurements. HoL wrote the manuscript, XC, JH, ZL, and XW modified the manuscript. All the authors have read and approved the publication of the manuscript.

## Conflict of Interest Statement

The authors declare that the research was conducted in the absence of any commercial or financial relationships that could be construed as a potential conflict of interest. The reviewer Q-YY declared a shared affiliation, though no other collaboration, with several of the authors HoL, ZL to the handling Editor.

## Footnotes

1 http://taylor0.biology.ucla.edu/structureHarvester/

2 www.omicshare.com/tools

## Supplementary Material

FIGURE S1 The distribution of SBR, SD, SS, and LC across 472 rapeseed accessions in five environments. **(A)**, **(D)**, **(H)**, **(L)**, and **(P)** the distribution of SBR in E1, E2, E3, E4, and E5, respectively. **(B)**, **(E)**, **(I)**, **(M)**, and **(Q)** the distribution of SD in E1, E2, E3, E4, and E5, respectively. **(C)**, **(F)**, **(J)**, **(N)**, and **(R)** the distribution of SS in E1, E2, E3, E4, and E5, respectively. **(G)**, **(K)**, **(O)**, and **(S)** the distribution of LC in E2, E3, E4, and E5, respectively. SBR, stem breaking resistance **(N)**; SD, stem diameter (mm); SS, stem strength (N/mm^2^); LC, lodging coefficient; E1, Wuhan in 2015; E2, Yangluo in 2015; E3, Wuhan in 2016; E4, Yangluo in 2016; and E5, Changsha in 2016.

FIGURE S2 Distribution of pairwise relative kinship. Only kinship coefficients of 0–0.5 are shown.

FIGURE S3 Venn diagram of differentially expressed genes (DEGs) identified by RNA-seq. FH, High-SBR during Flowering; FL, Low-SBR during Flowering; SH, High-SBR during Silique developing; SL, Low-SBR during Silique developing. The “up” and “down” indicate up-regulated expression and down-regulated expression, respectively.

FIGURE S4 GO enrichment analysis in molecular function for “green” module. The top 15 of pathway enrichment are shown.

FIGURE S5 Expression patterns of five DEGs. The bar plot shows the relative expression level determined by qRT-PCR. The heat map below the bar plot represents the expression level that was normalized based on FPKM from RNA-seq. FH, High-SBR during Flowering; FL, Low-SBR during Flowering; SH, High-SBR during Silique developing; SL, Low-SBR during Silique developing. Error bars, standard deviation; ^∗^ and ^∗∗^ above the bar represent significant a difference at 5 and 1% level, respectively.

TABLE S1 List of 472 rapeseed accessions used for association mapping.

TABLE S2 ANOVA and broad-sense heritability for stem lodging-related traits.

TABLE S3 List of SNPs associated significantly with stem lodging-related traits.

TABLE S4 The loci significantly associated with stem lodging-related traits.

TABLE S5 Summary of reads generated by RNA-seq.

TABLE S6 List of 8850 genes assigned into 25 different modules and their corresponding FPKM value across 12 samples.

TABLE S7 List of candidate genes for stem lodging-related traits.

TABLE S8 Primers used for qRT-PCR.

## References

[B1] Altschul S. F. Gish W. Miller W. Myers E. W. Lipman D. J. (1990). Basic local alignment search tool. *J. Mol. Biol.* 215 403–410. 10.1016/S0022-2836(05)80360-2 2231712

[B2] Anders S. Pyl P. T. Huber W. (2015). HTSeq–a Python framework to work with high-throughput sequencing data. *Bioinformatics* 31 166–169. 10.1093/bioinformatics/btu638 25260700 PMC4287950

[B3] Baker C. J. Sterling M. Berry P. (2014). A generalised model of crop lodging. *J. Theor. Biol.* 363 1–12. 10.1016/j.jtbi.2014.07.032 25109589

[B4] Barrett J. C. Fry B. Maller J. Daly M. J. (2005). Haploview: analysis and visualization of LD and haplotype maps. *Bioinformatics* 21 263–265. 10.1093/bioinformatics/bth457 15297300

[B5] Berry P. M. (2013). “Lodging resistance in cereals in sustainable food production,” in *Sustainable Food Production*, eds Christou P. Savin R. Costa-Pierce B. A. Misztal I. Whitelaw C. B. A. (Berlin: Springer), 1096–1110.

[B6] Bischoff V. Nita S. Neumetzler L. Schindelasch D. Urbain A. Eshed R. (2010). TRICHOME BIREFRINGENCE and its homolog AT5G01360 encode plant-specific DUF231 proteins required for cellulose biosynthesis in Arabidopsis. *Plant Physiol.* 153 590–602. 10.1104/pp.110.153320 20388664 PMC2879772

[B7] Bradbury P. J. Zhang Z. Kroon D. E. Casstevens T. M. Ramdoss Y. Buckler E. S. (2007). TASSEL: software for association mapping of complex traits in diverse samples. *Bioinformatics* 23 2633–2635. 10.1093/bioinformatics/btm308 17586829

[B8] Cericola F. Jahoor A. Orabi J. Andersen J. R. Janss L. L. Jensen J. (2017). Optimizing training population size and genotyping strategy for genomic prediction using association study results and pedigree information. A case of study in advanced wheat breeding lines. *PLoS One* 12:e0169606. 10.1371/journal.pone.0169606 28081208 PMC5231327

[B9] Chalhoub B. Denoeud F. Liu S. Parkin I. A. Tang H. Wang X. (2014). Early allopolyploid evolution in the post-Neolithic *Brassica napus* oilseed genome. *Science* 345 950–953. 10.1126/science.1253435 25146293

[B10] Chen B. Xu K. Li J. Li F. Qiao J. Li H. (2014). Evaluation of yield and agronomic traits and their genetic variation in 488 global collections of *Brassica napus* L. *Genet. Resour. Crop Evol.* 61 979–999. 10.1007/s10722-014-0091-8

[B11] Chen H. Shan Z. Sha A. Wu B. Yang Z. Chen S. (2011). Quantitative trait loci analysis of stem strength and related traits in soybean. *Euphytica* 179 485–497. 10.1007/s10681-011-0382-5

[B12] Chiniquy D. Varanasi P. Oh T. Harholt J. Katnelson J. Singh S. (2013). Three novel rice genes closely related to the Arabidopsis IRX9, IRX9L, and IRX14 genes and their roles in xylan biosynthesis. *Front. Plant Sci.* 4:83. 10.3389/fpls.2013.00083 23596448 PMC3622038

[B13] Dixit S. Grondin A. Lee C. R. Henry A. Olds T. M. Kumar A. (2015). Understanding rice adaptation to varying agro-ecosystems: trait interactions and quantitative trait loci. *BMC Genet.* 16:86. 10.1186/s12863-015-0249-1 26243626 PMC4526302

[B14] Earl D. A. Vonholdt B. M. (2012). STRUCTURE HARVESTER: a website and program for visualizing STRUCTURE output and implementing the Evanno method. *Conserv. Genet. Resour.* 4 359–361. 10.1007/s12686-011-9548-7

[B15] Evanno G. Regnaut S. Goudet J. (2005). Detecting the number of clusters of individuals using the software STRUCTURE: a simulation study. *Mol. Ecol.* 14 2611–2620. 10.1111/j.1365-294X.2005.02553.x 15969739

[B16] Fagard M. Desnos T. Desprez T. Goubet F. Refregier G. Mouille G. (2000). PROCUSTE1 encodes a cellulose synthase required for normal cell elongation specifically in roots and dark-grown hypocotyls of arabidopsis. *Plant Cell* 12 2409–2423. 10.1105/tpc.12.12.2409 11148287 PMC102227

[B17] Hématy K. Sado P.-E. Van Tuinen A. Rochange S. Desnos T. Balzergue S. (2007). A receptor-like kinase mediates the response of arabidopsis cells to the inhibition of cellulose synthesis. *Curr. Biol.* 17 922–931. 10.1016/j.cub.2007.05.018 17540573

[B18] Hua S. Zhang Y. Yu H. Lin B. Ding H. Zhang D. (2014). Paclobutrazol application effects on plant height, seed yield and carbohydrate metabolism in Canola. *Int. J. Agric. Biol.* 16 471–479.

[B19] Islam M. S. Peng S. B. Visperas R. M. Ereful N. Bhuiya M. S. U. Julfiquar A. W. (2007). Lodging-related morphological traits of hybrid rice in a tropical irrigated ecosystem. *Field Crop Res.* 101 240–248. 10.1016/j.fcr.2006.12.002

[B20] Jakobsson M. Rosenberg N. A. (2007). CLUMPP: a cluster matching and permutation program for dealing with label switching and multimodality in analysis of population structure. *Bioinformatics* 23 1801–1806. 10.1093/bioinformatics/btm233 17485429

[B21] Kashiwagi T. Ishimaru K. (2004). Identification and functional analysis of a locus for improvement of lodging resistance in rice. *Plant Physiol.* 134 676–683. 10.1104/pp.103.029355 14739343 PMC344543

[B22] Kendall S. L. Holmes H. White C. A. Clarke S. M. Berry P. M. (2017). Quantifying lodging-induced yield losses in oilseed rape. *Field Crop Res.* 211 106–113. 10.1016/j.fcr.2017.06.013

[B23] Kim D. Langmead B. Salzberg S. L. (2015). HISAT: a fast spliced aligner with low memory requirements. *Nat. Methods* 12 357–360. 10.1038/nmeth.3317 25751142 PMC4655817

[B24] Kobayashi Y. Sadhukhan A. Tazib T. Nakano Y. Kusunoki K. Kamara M. (2016). Joint genetic and network analyses identify loci associated with root growth under NaCl stress in *Arabidopsis thaliana*. *Plant Cell Environ.* 39 918–934. 10.1111/pce.12691 26667381

[B25] Kuai J. Sun Y. Zhou M. Zhang P. Zuo Q. Wu J. (2016). The effect of nitrogen application and planting density on the radiation use efficiency and the stem lignin metabolism in rapeseed (*Brassica napus* L.). *Field Crop Res.* 199 89–98. 10.1016/j.fcr.2016.09.025

[B26] Kumar M. Turner S. (2015). Plant cellulose synthesis: CESA proteins crossing kingdoms. *Phytochemistry* 112 91–99. 10.1016/j.phytochem.2014.07.009 25104231

[B27] Kumar S. Stecher G. Tamura K. (2016). MEGA7: molecular evolutionary genetics analysis version 7.0 for bigger datasets. *Mol. Biol. Evol.* 33 1870–1874. 10.1093/molbev/msw054 27004904 PMC8210823

[B28] Langfelder P. Horvath S. (2008). WGCNA: an R package for weighted correlation network analysis. *BMC Bioinformatics* 9:559. 10.1186/1471-2105-9-559 19114008 PMC2631488

[B29] Lee C. Teng Q. Zhong R. Ye Z. (2011). The four Arabidopsis reduced wall acetylation genes are expressed in secondary wall-containing cells and required for the acetylation of xylan. *Plant Cell Physiol.* 52 1289–1301. 10.1093/pcp/pcr075 21673009

[B30] Lefebvre V. Fortabat M. N. Ducamp A. North H. M. Maia-Grondard A. Trouverie J. (2011). ESKIMO1 disruption in Arabidopsis alters vascular tissue and impairs water transport. *PLoS One* 6:e16645. 10.1371/journal.pone.0016645 21408051 PMC3052256

[B31] Li F. Chen B. Xu K. Gao G. Yan G. Qiao J. (2016). A genome-wide association study of plant height and primary branch number in rapeseed (*Brassica napus*). *Plant Sci.* 242 169–177. 10.1016/j.plantsci.2015.05.012 26566834

[B32] Li F. Xie G. Huang J. Zhang R. Li Y. Zhang M. (2017). OsCESA9 conserved-site mutation leads to largely enhanced plant lodging resistance and biomass enzymatic saccharification by reducing cellulose DP and crystallinity in rice. *Plant Biotechnol. J.* 15 1093–1104. 10.1111/pbi.12700 28117552 PMC5552474

[B33] Li F. Chen B. Xu K. Wu J. Song W. Bancroft I. (2014). Genome-wide association study dissects the genetic architecture of seed weight and seed quality in rapeseed (*Brassica napus* L.). *DNA Res.* 21 355–367. 10.1093/dnares/dsu002 24510440 PMC4131830

[B34] Li S. Bashline L. Lei L. Gu Y. (2014). Cellulose synthesis and its regulation. *Arabidopsis Book.* 12:e0169. 10.1199/tab.0169 24465174 PMC3894906

[B35] Li Y. Gu H. Qi C. K. (2014). QTL mapping for lodging resistance and its related traits by RIL population of *Brassica napus* L. *Chin. J. Oil Crop Sci.* 36 10–17. 10.7505/j.issn.1007-9084.2014.01.002

[B36] Liu K. Muse S. V. (2005). PowerMarker: an integrated analysis environment for genetic marker analysis. *Bioinformatics* 21 2128–2129. 10.1093/bioinformatics/bti282 15705655

[B37] Merk H. L. Yarnes S. C. Van Deynze A. Tong N. K. Menda N. Mueller L. A. (2012). Trait diversity and potential for selection indices based on variation among regionally adapted processing tomato germplasm. *J. Am. Soc. Hortic. Sci.* 137 427–437.

[B38] Nei M. (1972). Genetic distance between populations. *Am. Nat.* 106 283–292. 10.1086/282771

[B39] Okushima Y. Mitina I. Quach H. L. Theologis A. (2005). AUXIN RESPONSE FACTOR 2 (ARF2): a pleiotropic developmental regulator. *Plant J.* 43 29–46. 10.1111/j.1365-313X.2005.02426.x 15960614

[B40] Peiffer J. A. Flint-Garcia S. A. De Leon N. Mcmullen M. D. Kaeppler S. M. Buckler E. S. (2013). The genetic architecture of maize stalk strength. *PLoS One* 8:e67066. 10.1371/journal.pone.0067066 23840585 PMC3688621

[B41] Peng X. (2012). *The Selection of Lodging Indicators and Mapping QTL for Lodging in Brassica napus L.* Master’s thesis, Southwest University, Chongqing.

[B42] Porth I. Klapste J. Skyba O. Hannemann J. Mckown A. D. Guy R. D. (2013). Genome-wide association mapping for wood characteristics in Populus identifies an array of candidate single nucleotide polymorphisms. *New Phytol.* 200 710–726. 10.1111/nph.12422 23889164

[B43] Pritchard J. K. Stephens M. Donnelly P. (2000). Inference of population structure using multilocus genotype data. *Genetics* 155 945–959. 10835412 10.1093/genetics/155.2.945 PMC1461096

[B44] Purcell S. Neale B. Todd-Brown K. Thomas L. Ferreira M. A. Bender D. (2007). PLINK: a tool set for whole-genome association and population-based linkage analyses. *Am. J. Hum. Genet.* 81 559–575. 10.1086/519795 17701901 PMC1950838

[B45] Rautengarten C. Ebert B. Herter T. Petzold C. J. Ishii T. Mukhopadhyay A. (2011). The interconversion of UDP-arabinopyranose and UDP-arabinofuranose is indispensable for plant development in Arabidopsis. *Plant Cell* 23 1373–1390. 10.1105/tpc.111.083931 21478444 PMC3101560

[B46] Rosenberg N. A. (2004). DISTRUCT: a program for the graphical display of population structure. *Mol. Ecol. Notes* 4 137–138. 10.1046/j.1471-8286.2003.00566.x

[B47] Samuga A. Joshi C. P. (2004). Differential expression patterns of two new primary cell wall-related cellulose synthase cDNAs, PtrCesA6 and PtrCesA7 from aspen trees. *Gene* 334 73–82. 10.1016/j.gene.2004.02.057 15256257

[B48] Shah A. N. Tanveer M. Rehman A. U. Anjum S. A. Iqbal J. Ahmad R. (2017). Lodging stress in cereal-effects and management: an overview. *Environ. Sci. Pollut. Res. Int.* 24 5222–5237. 10.1007/s11356-016-8237-1 28025787

[B49] Shannon P. Markiel A. Ozier O. Baliga N. S. Wang J. T. Ramage D. (2003). Cytoscape: a software environment for integrated models of biomolecular interaction networks. *Genome Res.* 13 2498–2504. 10.1101/gr.1239303 14597658 PMC403769

[B50] Shin J.-H. Blay S. Graham J. Mcneney B. (2006). LDheatmap: an R function for graphical display of pairwise linkage disequilibria between single nucleotide polymorphisms. *J. Stat. Softw.* 16 1–10. 10.18637/jss.v016.c03

[B51] Sibout R. Eudes A. Mouille G. Pollet B. Lapierre C. Jouanin L. (2005). CINNAMYL ALCOHOL DEHYDROGENASE-C and -D are the primary genes involved in lignin biosynthesis in the floral stem of Arabidopsis. *Plant Cell* 17 2059–2076. 10.1105/tpc.105.030767 15937231 PMC1167552

[B52] Somerville C. (2006). Cellulose synthesis in higher plants. *Annu. Rev. Cell Dev. Biol.* 22 53–78. 10.1146/annurev.cellbio.22.022206.160206 16824006

[B53] Stewart D. W. Costa C. Dwyer L. M. Smith D. L. Hamilton R. I. Ma B. L. (2003). Canopy structure, light interception, and photosynthesis in maize. *Agron. J.* 95 1465–1474. 10.2134/agronj2003.1465

[B54] Sun Z. Wang X. Liu Z. Gu Q. Zhang Y. Li Z. (2017). Genome-wide association study discovered genetic variation and candidate genes of fibre quality traits in *Gossypium hirsutum* L. *Plant Biotechnol. J.* 15 982–996. 10.1111/pbi.12693 28064470 PMC5506648

[B55] Tumino G. Voorrips R. E. Rizza F. Badeck F. W. Morcia C. Ghizzoni R. (2016). Population structure and genome-wide association analysis for frost tolerance in oat using continuous SNP array signal intensity ratios. *Theor. Appl. Genet.* 129 1711–1724. 10.1007/s00122-016-2734-y 27318699 PMC4983288

[B56] Urbanowicz B. R. Pena M. J. Moniz H. A. Moremen K. W. York W. S. (2014). Two Arabidopsis proteins synthesize acetylated xylan in vitro. *Plant J.* 80 197–206. 10.1111/tpj.12643 25141999 PMC4184958

[B57] Wang N. Chen B. Xu K. Gao G. Li F. Qiao J. (2016). Association mapping of flowering time QTLs and insight into their contributions to rapeseed growth habits. *Front. Plant Sci.* 7:338. 10.3389/fpls.2016.00338 27047517 PMC4805649

[B58] Wei D. Cui Y. He Y. Xiong Q. Qian L. Tong C. (2017). A genome-wide survey with different rapeseed ecotypes uncovers footprints of domestication and breeding. *J. Exp. Bot.* 68 4791–4801. 10.1093/jxb/erx311 28992309 PMC5853444

[B59] Wei L. Jian H. Lu K. Filardo F. Yin N. Liu L. (2016). Genome-wide association analysis and differential expression analysis of resistance to Sclerotinia stem rot in *Brassica napus*. *Plant Biotechnol. J.* 14 1368–1380. 10.1111/pbi.12501 26563848 PMC11389038

[B60] Wei L. Jian H. Lu K. Yin N. Wang J. Duan X. (2017). Genetic and transcriptomic analyses of lignin- and lodging-related traits in *Brassica napus*. *Theor. Appl. Genet.* 130 1961–1973. 10.1007/s00122-017-2937-x 28634809

[B61] Wei T. Simko V. (2016). *corrplot: Visualization of a Correlation Matrix. R package Version 0.77.* Available at: https://CRAN.R-project.org/package=corrplot

[B62] Wen Z. Boyse J. F. Song Q. Cregan P. B. Wang D. (2015). Genomic consequences of selection and genome-wide association mapping in soybean. *BMC Genomics* 16:671. 10.1186/s12864-015-1872-y 26334313 PMC4559069

[B63] Wieczorek K. Golecki B. Gerdes L. Heinen P. Szakasits D. Durachko D. M. (2006). Expansins are involved in the formation of nematode-induced syncytia in roots of *Arabidopsis thaliana*. *Plant J.* 48 98–112. 10.1111/j.1365-313X.2006.02856.x 16942607

[B64] Xu L. Hu K. Zhang Z. Guan C. Chen S. Hua W. (2016). Genome-wide association study reveals the genetic architecture of flowering time in rapeseed (*Brassica napus* L.). *DNA Res.* 23 43–52. 10.1093/dnares/dsv035 26659471 PMC4755526

[B65] Yan G. Lv X. Gao G. Li F. Li J. Qiao J. (2016). Identification and characterization of a glyoxalase I gene in a rapeseed cultivar with seed thermotolerance. *Front. Plant Sci.* 7:150. 10.3389/fpls.2016.00150 26909093 PMC4754733

[B66] Yang J. Lee S. H. Goddard M. E. Visscher P. M. (2011). GCTA: a tool for genome-wide complex trait analysis. *Am. J. Hum. Genet.* 88 76–82. 10.1016/j.ajhg.2010.11.011 21167468 PMC3014363

[B67] Yuan Y. Teng Q. Zhong R. Haghighat M. Richardson E. A. Ye Z. H. (2016). Mutations of Arabidopsis TBL32 and TBL33 affect xylan acetylation and secondary wall deposition. *PLoS One* 11:e0146460. 10.1371/journal.pone.0146460 26745802 PMC4712945

[B68] Yuan Y. Teng Q. Zhong R. Ye Z. H. (2013). The Arabidopsis DUF231 domain-containing protein ESK1 mediates 2-O- and 3-O-acetylation of xylosyl residues in xylan. *Plant Cell Physiol.* 54 1186–1199. 10.1093/pcp/pct070 23659919

[B69] Zhang B. Deng L. Qian Q. Xiong G. Zeng D. Li R. (2009). A missense mutation in the transmembrane domain of CESA4 affects protein abundance in the plasma membrane and results in abnormal cell wall biosynthesis in rice. *Plant Mol. Biol.* 71 509. 10.1007/s11103-009-9536-4 19697141

[B70] Zhang B. Horvath S. (2005). A general framework for weighted gene co-expression network analysis. *Stat. Appl. Genet. Mol. Biol.* 4:Article17. 10.2202/1544-6115.1128 16646834

[B71] Zhong R. Pena M. J. Zhou G. K. Nairn C. J. Wood-Jones A. Richardson E. A. (2005). Arabidopsis fragile fiber8, which encodes a putative glucuronyltransferase, is essential for normal secondary wall synthesis. *Plant Cell* 17 3390–3408. 10.1105/tpc.105.035501 16272433 PMC1315377

