# Supplementary figures and images for: An Integration of Genome-Wide Association Study and Gene Co-expression Network Analysis Identifies Candidate Genes of Stem Lodging-Related Traits in Brassica napus
Source: Front Plant Sci. 2018 Jun 12;9:796. doi: 10.3389/fpls.2018.00796 (PMC6006280; doi:10.3389/fpls.2018.00796)

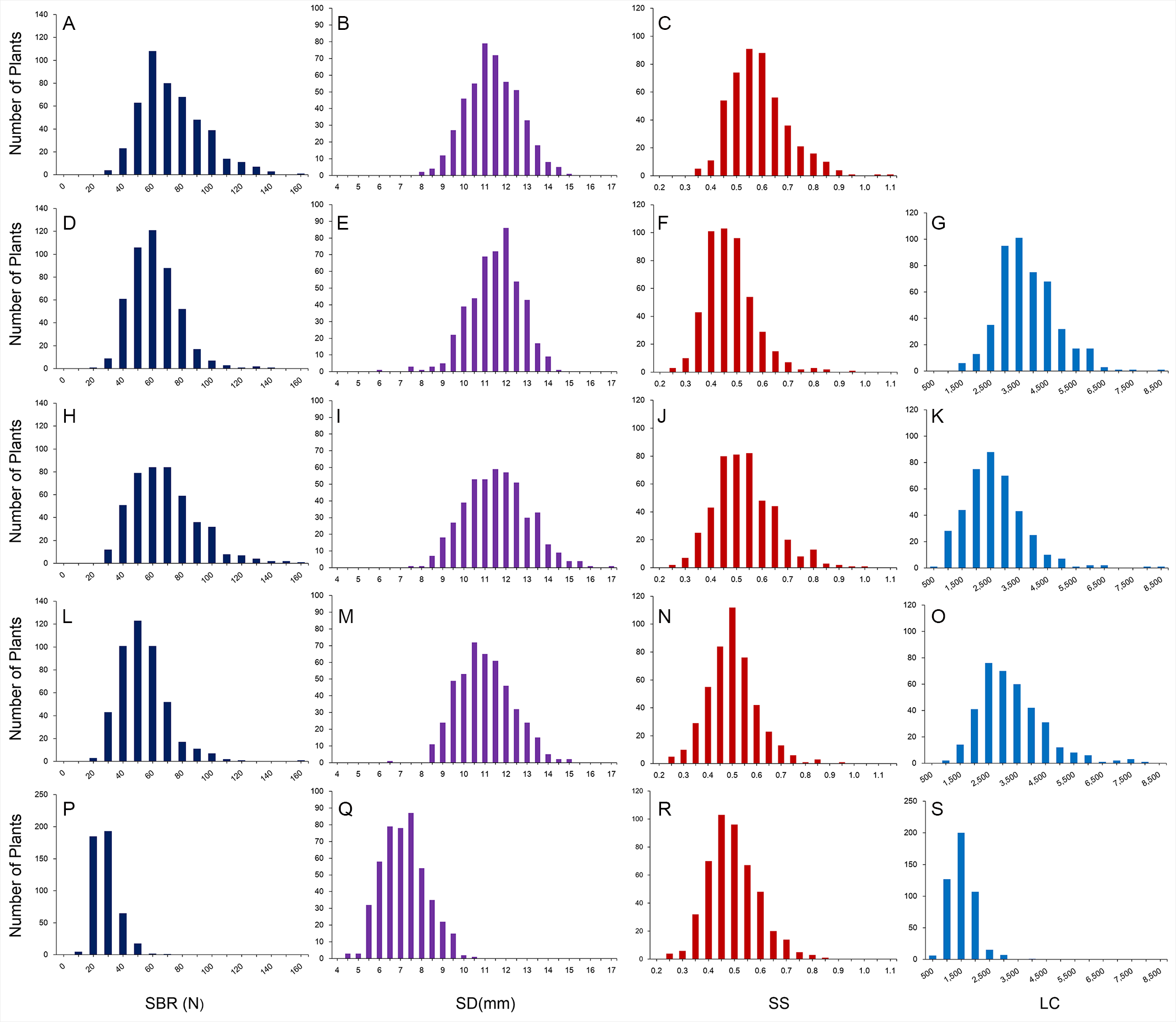

Supplement: FIGURE S1 — The distribution of SBR, SD, SS, and LC across 472 rapeseed accessions in five environments. (A), (D), (H), (L), and (P) the distribution of SBR in E1, E2, E3, E4, and E5, respectively. (B), (E), (I), (M), and (Q) the distribution of SD in E1, E2, E3, E4, and E5, respectively. (C), (F), (J), (N), and (R) the distribution of SS in E1, E2, E3, E4, and E5, respectively. (G), (K), (O), and (S) the distribution of LC in E2, E3, E4, and E5, respectively. SBR, stem breaking resistance (N); SD, stem diameter (mm); SS, stem strength (N/mm2); LC, lodging coefficient; E1, Wuhan in 2015; E2, Yangluo in 2015; E3, Wuhan in 2016; E4, Yangluo in 2016; and E5, Changsha in 2016. [file Image_1.TIF]

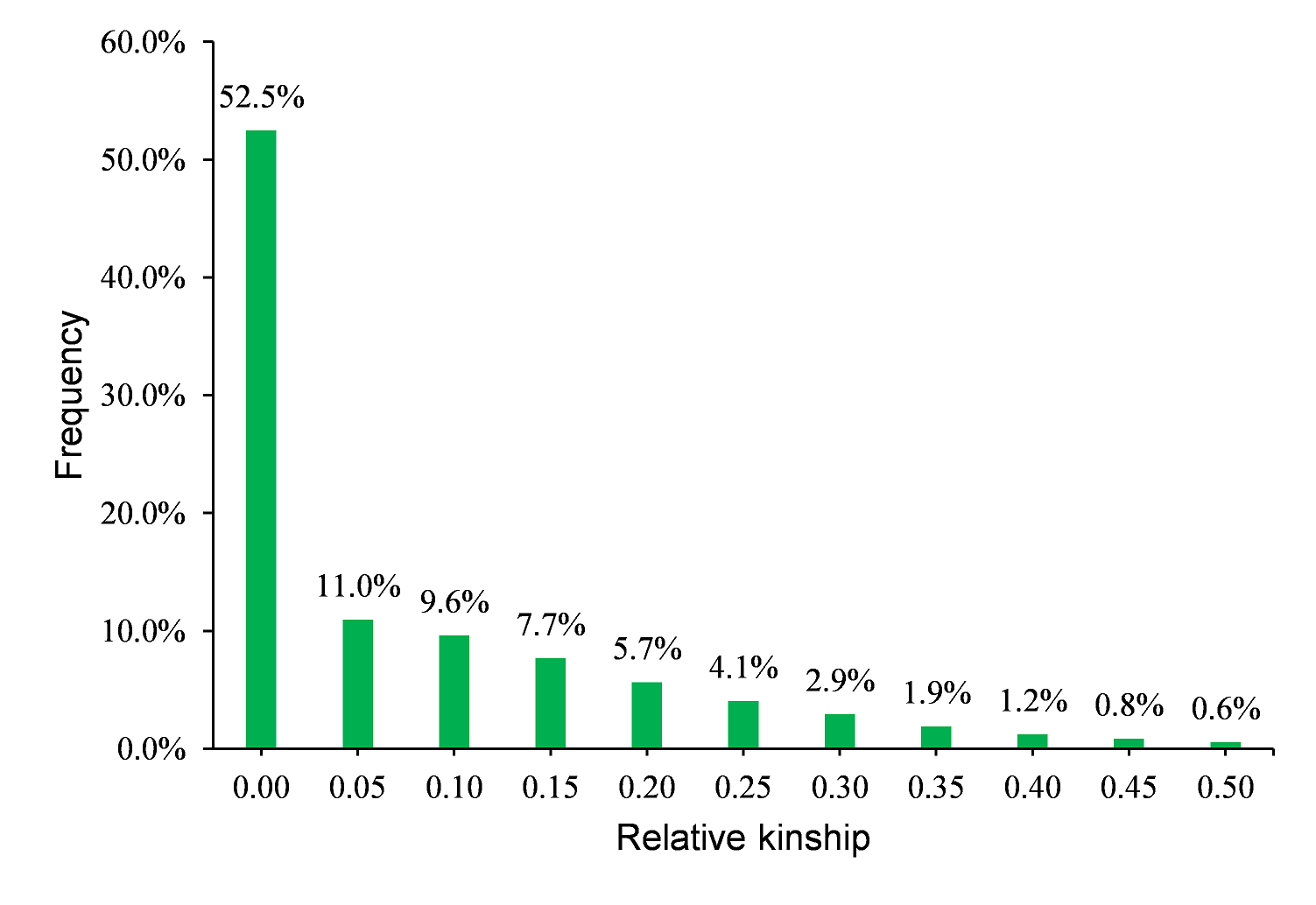

Supplement: FIGURE S2 — Distribution of pairwise relative kinship. Only kinship coefficients of 0–0.5 are shown. [file Image_2.TIF]

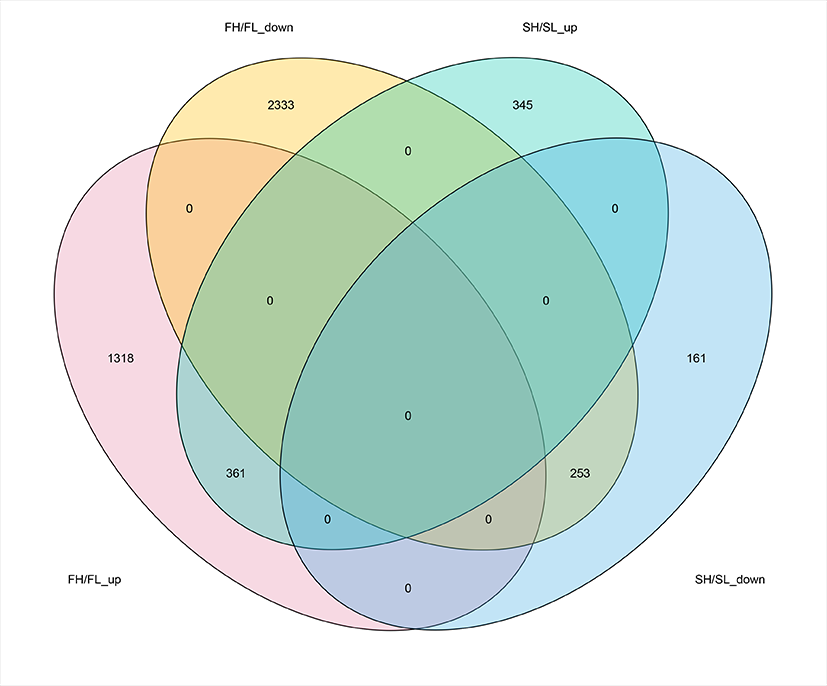

Supplement: FIGURE S3 — Venn diagram of differentially expressed genes (DEGs) identified by RNA-seq. FH, High-SBR during Flowering; FL, Low-SBR during Flowering; SH, High-SBR during Silique developing; SL, Low-SBR during Silique developing. The “up” and “down” indicate up-regulated expression and down-regulated expression, respectively. [file Image_3.TIF]

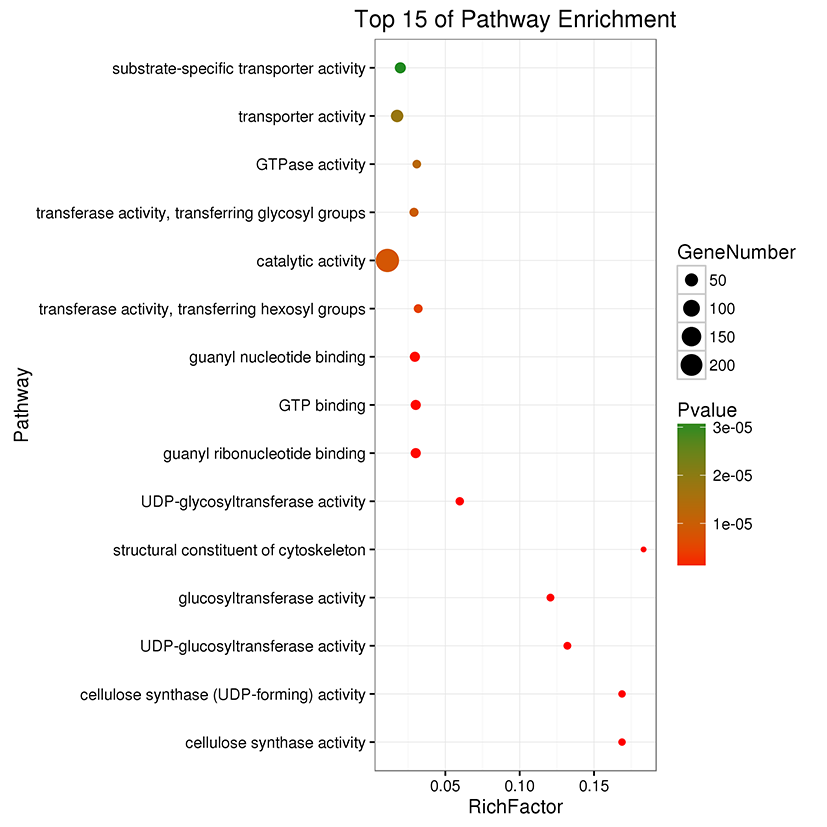

Supplement: FIGURE S4 — GO enrichment analysis in molecular function for “green” module. The top 15 of pathway enrichment are shown. [file Image_4.TIF]

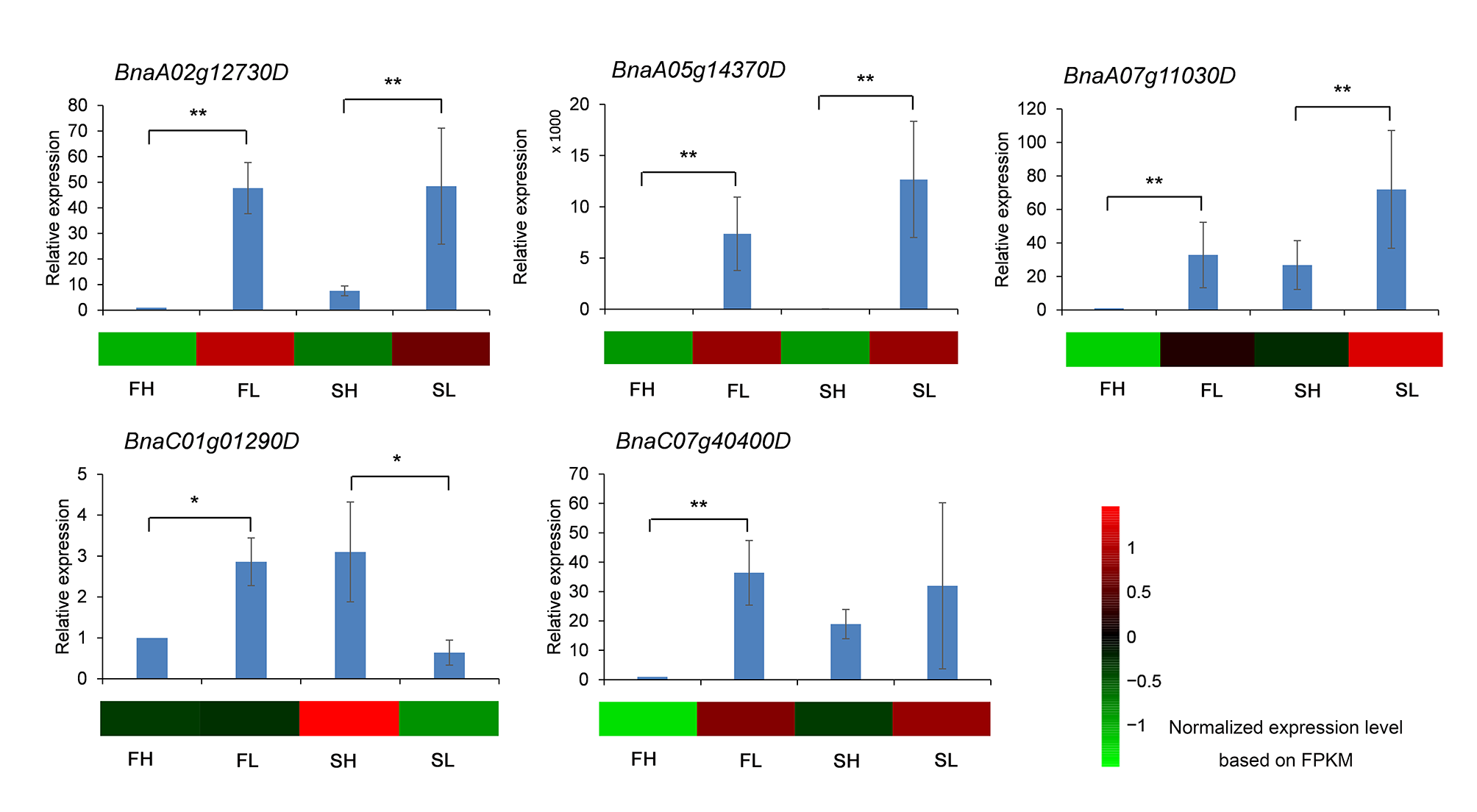

Supplement: FIGURE S5 — Expression patterns of five DEGs. The bar plot shows the relative expression level determined by qRT-PCR. The heat map below the bar plot represents the expression level that was normalized based on FPKM from RNA-seq. FH, High-SBR during Flowering; FL, Low-SBR during Flowering; SH, High-SBR during Silique developing; SL, Low-SBR during Silique developing. Error bars, standard deviation; ∗ and ∗∗ above the bar represent significant a difference at 5 and 1% level, respectively. [file Image_5.TIF]
